# Effects of Subanesthetic Oromucosal Dexmedetomidine on Sleep in Humans: A Randomized, Controlled Pharmacokinetics–Pharmacodynamics Study

**DOI:** 10.1097/ALN.0000000000005314

**Published:** 2024-11-27

**Authors:** Laura K. Schnider, Marta Ratajczak, Rafael Wespi, Jacqueline G. Kientsch, Francesco Bavato, Laurenz Marten, Jonas Kost, Maxim Puchkov, Corinne Eicher, Martina Boxler, Clarissa D. Voegel, Oliver G. Bosch, Eus van Someren, Dario A. Dornbierer, Hans-Peter Landolt

**Affiliations:** 1 Institute of Pharmacology and Toxicology, University of Zurich, Zurich, Switzerland.; 2 Institute of Pharmacology and Toxicology, University of Zurich, Zurich, Switzerland.; 3 Institute of Pharmacology and Toxicology, University of Zurich, Zurich, Switzerland.; 4 Institute of Pharmacology and Toxicology, University of Zurich, Zurich, Switzerland.; 5 Department of Adult Psychiatry and Psychotherapy, University Hospital of Psychiatry, University of Zurich, Zurich, Switzerland.; 6 Department of Adult Psychiatry and Psychotherapy, University Hospital of Psychiatry, University of Zurich, Zurich, Switzerland.; 7 Department of Pharmaceutical Sciences, University of Basel, Basel, Switzerland.; 8 Department of Pharmaceutical Sciences, University of Basel, Basel, Switzerland.; 9 Institute of Pharmacology & Toxicology, and Department of Adult Psychiatry and Psychotherapy, University Hospital of Psychiatry, University of Zurich, Zurich, Switzerland.; 10 Institute of Forensic Medicine, University of Zurich, Zurich, Switzerland.; 11 Center for Forensic Hair Analytics, Zurich Institute of Forensic Medicine, University of Zurich, Zurich, Switzerland.; 12 Department of Adult Psychiatry and Psychotherapy, University Hospital of Psychiatry, University of Zurich, Zurich, Switzerland.; 13 Department of Sleep and Cognition, Netherlands Institute for Neuroscience, Amsterdam, The Netherlands.; 14 Institute of Pharmacology and Toxicology, and Department of Adult Psychiatry and Psychotherapy, University Hospital of Psychiatry, University of Zurich, Zurich, Switzerland.; 15 Institute of Pharmacology and Toxicology, and Sleep and Health Zurich, University of Zurich, Zurich, Switzerland.

## Abstract

**Background:**

The locus coeruleus noradrenergic system may provide a potential new target for pharmacologic insomnia treatment, particularly in patients suffering from elevated distress. The selective α_2_-noradrenergic agonist dexmedetomidine attenuates locus coeruleus activity in subanesthetic doses, yet no adequate nonparental delivery systems of dexmedetomidine are currently available. To examine the feasibility of oromucosal dexmedetomidine administration, the authors developed two distinct—one sublingual and one buccal—oromucosal, fast-disintegrating dexmedetomidine formulas tailored for self-administration. Here, the authors established the formulas’ pharmacokinetic and pharmacodynamic profiles.

**Methods:**

In a pilot study (sublingual formulation; n = 8 good sleepers) and a main study (buccal formulation; n = 17 poor sleepers), each following a randomized, double-blind, placebo-controlled crossover design, the authors investigated subanesthetic doses (20 and 40 µg) of the two formulas. They complemented the pharmacokinetic assessments with all-night polysomnography, nocturnal cortisol and melatonin measurements, assessments of cardiovascular functions during and after sleep, cortisol awakening response, and postawakening examination of subjective state and vigilance.

**Results:**

Particularly buccal dexmedetomidine was rapidly absorbed and exhibited excellent dose proportionality with minimal between-subject variation in exposure. In poor sleepers, 40 µg buccal dexmedetomidine shortened the sleep latency by 11.5 min, increased the time spent in non–rapid eye movement sleep by 37.2 min, and elevated non–rapid eye movement sleep electroencephalographic slow-wave energy (0.75 to 4.0 Hz) in the first half of the night by roughly 23%. Rapid eye movement sleep latency was dose-dependently prolonged (20 µg, 55.0 min; 40 µg, 115.3 min). Nocturnal cortisol, melatonin and heart rate, and morning cortisol were not significantly affected by dexmedetomidine, nor did postawakening orthostatic regulation, subjective sleepiness and mood, and psychomotor vigilance differ among the conditions.

**Conclusions:**

The favorable pharmacokinetic and pharmacodynamic profile of oromucosal dexmedetomidine delivery warrants further dose-finding and clinical studies to establish the exact roles of α_2_ receptor agonism in pharmacologic sleep enhancement and as a possible novel mechanism to alleviate stress-related insomnia.

## Abstract 2

Buccal delivery of dexmedetomidine results in rapid absorption and good bioavailability. In poor sleepers, doses of 20 and 40 μg dexmedetomidine resulted in shortened sleep latency and increased non–rapid eye movement sleep without impairment of vigilance on awakening the next morning. Buccal dexmedetomidine delayed the onset of rapid eye movement sleep.

**Editor’s Perspective**

**What We Already Know about This Topic**

- Dexmedetomidine is known to enhance sleep-like states
- Because of variable absorption and high first-pass metabolism, nasal and oral formulations of dexmedetomidine produce inconsistent blood levels

**What This Article Tells Us That Is New**

- Buccal delivery of dexmedetomidine results in rapid absorption and good bioavailability
- In poor sleepers, doses of 20 and 40 μg of dexmedetomidine resulted in shortened sleep latency and increased non–rapid eye movement sleep without impairment of vigilance on awakening the next morning
- Buccal dexmedetomidine delayed the onset of rapid eye movement sleep

Insomnia is the second most prevalent neuropsychiatric disorder. Although they provide symptomatic relief, currently approved sleep medications do not promote physiologic sleep processes and are prone of unwanted effects like dependence, tolerance, and daytime impairments.^1,2^ Thus, there exists an unmet clinical need for novel pharmacotherapeutic approaches to restore physiologic sleep functions.

The locus coeruleus noradrenergic system may be a promising new target to ameliorate stress-induced sleep disturbances.^3^ The locus coeruleus represents the main source of brain norepinephrine synthesis and constitutes an integral part of the ascending wake-promoting system.^4^ By acting on specific α and β adrenoceptors, the norepinephrine also regulates essential waking functions including attention, arousal, memory, emotions, and cognition. The locus coeruleus activity is high in wakefulness, lower during non–rapid eye movement (NREM) sleep, and reaches near complete silence in rapid eye movement (REM) sleep.^5^ Because uniquely low norepinephrine levels are reached in REM sleep, consolidated REM sleep may be beneficial to normalize distress and negative affect across the night.^3,6^ Thus, it has been hypothesized that stable REM sleep optimally favors adaptive synaptic plasticity during replay of emotional memory traces in the limbic circuit, which is highly active in this sleep stage. Entering sleep with lingering arousal prevents the locus coeruleus shut-off required for sound sleep, and sustained locus coeruleus activity not only fragments REM sleep but also reduces deep NREM sleep. Such sleep disturbances are seen in many neuropsychiatric patients.^7,8^ In addition, restless sleep may not merely be a symptom of an underlying neuropsychiatric disease, but may itself play a crucial role in the pathogenesis and progression of the disorder. Given these mutual relationships, reducing locus coeruleus activity could provide a promising strategy to break the vicious circle of disturbed nocturnal sleep and affective disorders.

The highly selective α_2_-adrenoceptor agonist, dexmedetomidine, recently elicited interest as potential repurposing candidate to enhance sleep in various medical conditions.^9,10^ Dexmedetomidine activates inhibitory, presynaptic α_2_ receptors on the locus coeruleus and inhibits the release of norepinephrine.^11–13^ The compound is registered as IV anesthetic and widely used to optimize sedation and analgesia during surgery, with reduced risk of postoperative delirium.^14^ In patients undergoing emergency trauma surgery, low-dose dexmedetomidine prevented the development of postsurgery posttraumatic stress disorder (PTSD) symptoms and improved overall sleep quality.^15^ Furthermore, IV administration of dexmedetomidine improved sleep quality in patients with severe treatment-resistant insomnia.^16^

Because of pronounced first-pass metabolism,^17,18^ dexmedetomidine administration currently requires IV injection in controlled settings. These restraints hamper the possible use of dexmedetomidine as sleep medication at home. Previous attempts at noninvasive (*e.g.*, oral, nasal) dexmedetomidine delivery were characterized by low and highly variable absorption.^18,19^ To overcome these challenges, we developed innovative, fast-disintegrating, oromucosal formulations for sublingual or buccal dexmedetomidine administration. To establish the feasibility of oromucosal dexmedetomidine delivery, we conducted two randomized, placebo-controlled crossover trials and comprehensively tested the pharmacokinetics and sleep effects of two subanesthetic (20 and 40 µg) dexmedetomidine doses in a pilot study in healthy good sleepers and in the main study in poor sleepers reporting subclinical symptoms of insomnia.

## Materials and Methods

### Permission

The study was first submitted on July 31, 2020, for ethical approval by the Cantonal Ethics Committee of the Canton of Zurich (Swiss Association of Research Ethics Committees registration No. 2020-016114; principal investigator, Oliver G. Bosch, M.D.). Upon receipt of the approval, it was registered on ClinicalTrials.gov (identifier No. NCT04508166). All participants provided written informed consent according to the declaration of Helsinki.

### Study Participants

The pharmacokinetic and pharmacodynamic properties of oromucosal dexmedetomidine were investigated in two separate studies. We first conducted a pilot study in 8 healthy young men with good sleep quality (referred to as “good sleepers”) and a main study in 17 healthy young men reporting subclinical insomnia (“poor sleepers”). We did not perform an *a priori* sample size calculation and aimed for a sample size similar to previous studies of us and others addressing similar research questions.^18,20,21^ The sample size was approved by the ethics committee based upon the weighed potential risks and benefits. Apart from the study populations, the two studies also slightly differed in the galenic formulations and the exact routes of oromucosal dexmedetomidine application (see section "Study Drugs and Design"). The following inclusion criteria were required for enrollment in both studies: male sex, to avoid unrecognized pregnancy and the impact of the menstrual cycle on sleep physiology or hypothalamus-pituitary axis activity; age range, 18 to 35 yr; body mass index, 18.5 to 25 kg/m^2^; regular habitual sleep–wake rhythm (bedtime, 10:00 pm and 12:00 am); absence of somatic or psychiatric disorders; normal or corrected to normal vision; no acute or chronic medication intake; nonsmoking; no history of drug abuse (exception: occasional cannabis use); moderate alcohol (less than 5 U/week) and caffeine consumption (less than U/day); no crossing of more than 2 time zones within 30 days before study start. For inclusion in the second study, an Insomnia Severity Index score between 8 and 14 was required.

Before final enrollment as study participant, all volunteers completed a screening night in the sleep laboratory, to exclude sleep-related disorders such as sleep apnea (apnea-hypopnea index more than 5 h of sleep), periodic limb movements (more than 5 h of sleep), or sleep onset REM sleep episodes. Enrolled participants were required to abstain from illicit drugs, starting 2 weeks before the first experimental night until the end of the study (the day after the third experimental night). Intake of alcohol was not allowed within 24 h of the start of the experimental nights. Participants were also instructed to keep an individual, regular sleep–wake rhythm with 8 h time in bed during the entire study, starting 2 weeks before the first experimental night. The rhythm was chosen depending on the volunteers’ habitual bedtime. All included participants chose to keep a bedtime between 10:00 pm and 12:00 am and a corresponding rise time between 6:00 am and 8:00 am. On average, bedtime occurred at 11:24 pm ± 0.31 min. Adherence to the sleep–wake schedule was monitored with a rest–activity monitor (GENEActiv, Activinsights Ltd., United Kingdom) worn on the wrist of the nondominant arm and a sleep–wake diary. Fifteen minutes before bedtime, an evening questionnaire was completed daily throughout the entire study, which, on experimental nights, also served as the evaluation of the consumption of drugs, consumption of alcoholic or caffeinated beverages, and adherence to the regular sleep–wake rhythm.

The demographic characteristics of the study participants are reported in Supplemental Digital Content table S1 (https://links.lww.com/ALN/D763).

### Study Drugs and Design

#### Sublingual Orodispersible Tablet

For the study in the good sleepers’ group, a first sublingual orodispersible tablet formulation was developed by direct tablet compaction of dexmedetomidine–hydro-chloride with tricalcium phosphate as bulking agent and croscarmellose sodium as super-disintegrant.^22^ For this purpose, Dexdor injectable solution (100 µg/ml; Orion Pharm AG, Switzerland) was added in a rotovap flask to tricalcium phosphate powder (Galvita AG, Switzerland), and the mixture was dried under vacuum in a rotary evaporator (40°C; 2 h). Ac-Di-Sol (3%; cross-linked sodium carboxymethyl cellulose; FMC Biopolymer, USA) was added as super-disintegrant, and the blend was homogenized for 10 min in a Turbula mixer (Willy A. Bachofen AG, Switzerland). Then, 104.7 mg of the blend was filled into the 7-mm die and compacted with 1 mT. The placebo tablets were obtained accordingly, but Dexdor was replaced by saline 0.9% (B. Braun Medical AG, Switzerland) to mimic the slight salty taste of the Dexdor solution. All tablets were stored at room temperature under dry conditions (desiccator bag).

#### Buccal Orodispersible Tablet

For the study in the poor sleepers’ group, an improved buccal orodispersible tablet formulation was developed by freeze-drying for buccal delivery. Dextran FP40 (SERVA Electrophoresis GmbH, Germany) was used as bulking agent and dissolved in Dexdor injectable solution (100 µg/ml; Orion Pharm AG, Switzerland). The solution was then volumetrically filled into aluminum blister molds (0.2 ml/cavity) using an Eppendorf Micropipette (Eppendorf GmbH, Germany) and finally freeze-dried for 30 h, to yield lyophilized tablets with a strength of 20 µg each. The placebo melting tablets were obtained accordingly, but Dexdor was replaced by saline 0.9% (B. Braun Medical AG, Switzerland) to mimic the slight salty taste of the Dexdor solution. All tablets were stored at room temperature under dry conditions (desiccator bag).

We examined the pharmacokinetic and pharmacodynamic profiles of sublingual and buccal dexmedetomidine administration. The first study in the good sleepers (sublingual orodispersible tablet) mainly served as pilot study to evaluate the performance of oromucosal dexmedetomidine delivery, identify an adequate dose range, and determine preliminary effects on sleep physiology.

### Study Design

**Fig. 1.**
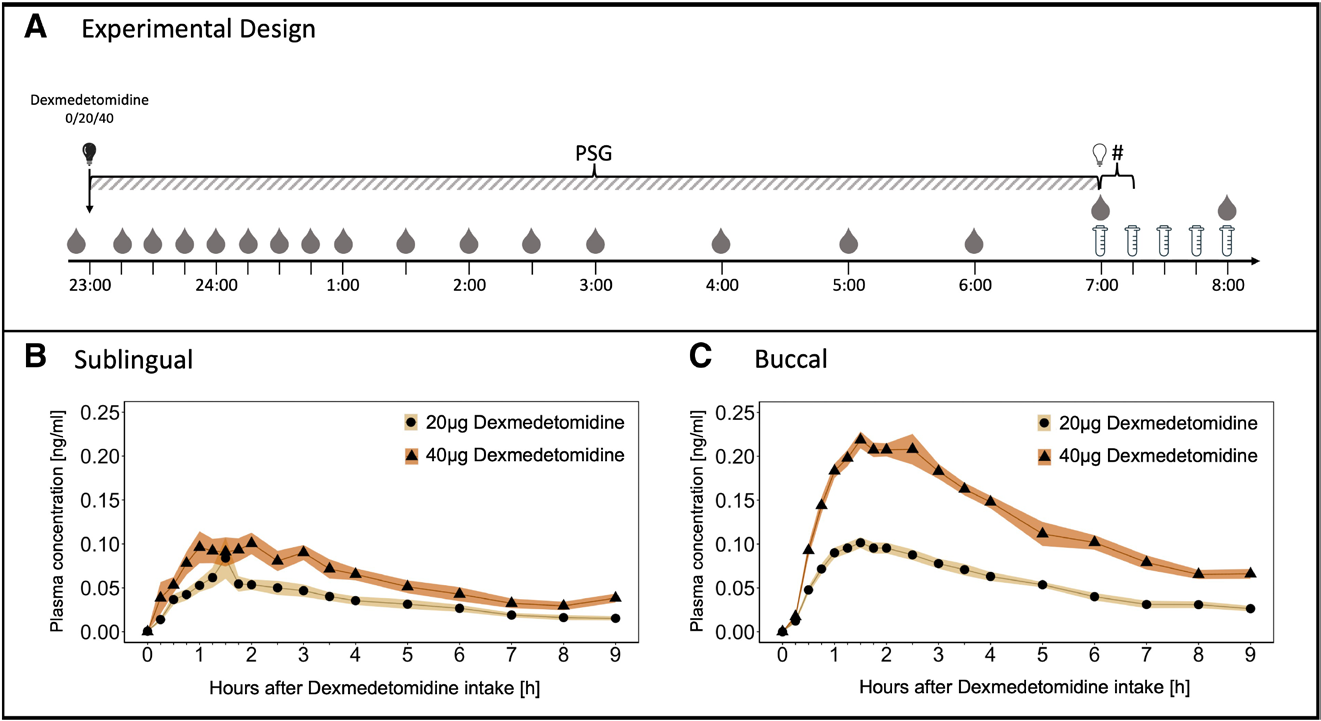
Experimental design and pharmacokinetic profiles of dexmedetomidine after sublingual and buccal orodispersible tablet intake. (*A*) Time points of blood draws (*drops*) , saliva sampling (*tubes*), and postawakening testing (#) are indicated on the x-axis. The study drugs (0/20/40 µg dexmedetomidine) were administered at 11:00 pm when lights were switched off (*black light bulb*) and the polysomnographic (PSG) recording was initiated (time point 0). The participants were awoken and lights were turned on at 7:00 am (time point 8). (*B*) Evolution of dexmedetomidine plasma concentration after sublingual dexmedetomidine intake (n = 8). (*C*) Evolution of dexmedetomidine plasma concentration after buccal dexmedetomidine intake (n = 17). *Yellow lines* connecting *black dots*: 20 µg dexmedetomidine. *Orange lines* connecting *black triangles*: 40 µg dexmedetomidine. *Yellow and orange shadings* indicate standard deviations.

Both studies adhered to a randomized, placebo-controlled, balanced, double-blind, crossover design. The study protocols consisted of one screening and three experimental nights (placebo, 20 and 40 μg dexmedetomidine) separated by a washout period of 7 days. Placebo and verum tablets were combined to yield total doses of 0 µg (2 × placebo), 20 µg (1 × 20 µg + 1 × placebo), and 40 µg (2 × 20 µg) dexmedetomidine. The assessment of the compound’s effect on neurobehavioral, emotional, cognitive, and endocrinological markers of sleep inertia followed immediately after awakening. To simplify descriptions and data presentations, we will refer to the scheduled 11:00 pm to 7:00 am time in bed with respect to the time points of the tasks, because most of the participants roughly followed this sleep–wake schedule. The details of the study design are illustrated in figure 1A.

For each study participant, there were six possible orders of the three experimental conditions. A member of the study team (H.-P.L.) who was not involved in participant enrollment or data collection and analyses prospectively employed an online dice (https://www.wuerfel.jetzt/) to produce a random drug allocation sequence for consecutive participants, separately for the pilot study and the main study. The blinding codes with the allocation sequences were locked in an office that is only accessible to this member of the study team. The sequentially numbered containers, each containing three small bags with two identical tablets, were kept in a locked safe in a locked room of the sleep laboratory. On each experimental condition, a different member of the study team administered the two prepacked tablets to each participant and supervised the intake. The sequence of the interventions was kept blind to all participants and all experimenters until the visual scoring of the sleep variables and the transcription of all data was completed and locked.

### Bioanalyses of Blood Plasma Samples

#### Blood Sampling

After drug intake at bedtime, study participants were allowed to sleep for 8 h in the soundproof and climatized bedrooms of the sleep laboratory. Blood samples were continuously collected from the left (in case of unsuccessful venipuncture from the right) antecubital vein *via* a venous catheter connected with Heidelberger plastic tube extensions to the adjacent room. Blood was drawn at baseline (30 min before drug intake) and 0.25, 0.5, 0.75, 1, 1.25, 1.5, 1.75, 2, 2.5, 3, 3.5, 4, 5, 6, 7, 8, and 9 h after drug administration (4 ml, BD Vacutainer EDTA [BD, Switzerland]), while keeping the disturbance of the participants’ sleep to a minimum. The peripheral venous access was kept patent *via* a constant drip (10 ml/h) of heparinized saline (1,000 U heparin in 0.9 g NaCl/dl; HEPARIN Bichsel, Bichsel AG, Switzerland). Blood samples were centrifuged for 5 min at 2,000 relative centrifugal force immediately after blood drawing. The resulting plasma samples were directly transferred to –20°C until final storage at –80°C.

#### Quantification of Dexmedetomidine

The dexmedetomidine was purchased from Sigma-Aldrich (USA), and medetomidine-^13^C-d_3_ was purchased from Cayman Chemical (USA) and used as internal standard. All chemicals used were of the highest purification grade available. A total of 600 µl plasma and 30 µl internal standard (8 ng/ml) were added to a tube. For calibrator and quality control samples, an additional 100 µl of the calibrator or quality control solutions were added. A liquid–liquid extraction using 1,000 µl ethyl acetate/butyl acetate (1:1 v/v) was performed by shaking the tubes for 10 min and centrifuging them at 12,000 rotations per minute for 5 min. A volume of 900 µl of the organic phase were transferred into a vial and evaporated to dryness under a gentle stream of nitrogen, before adding 50 µl of an eluent-mixture (85:15 v/v) for reconstitution. The samples were analyzed on an ultra-high performance liquid chromatography system (Thermo Fisher, USA), coupled to a linear Ion Trap Quadrupole Mass Spectrometer 5,500 (Sciex, Germany). The mass spectrometer was operated in positive electrospray ionization mode with multiple reaction monitoring. Three transitions were used for dexmedetomidine: 200.9 → 95.3, 200.9 → 68, and 200.9 → 41. The mobile phases of the ultra-high performance liquid chromatography system consisted of water (eluent A) and acetonitrile (eluent B), both containing 0.1% formic acid (v/v). The samples (2 μl) were injected using a Kinetex C18 column (100 × 2.1 mm, 1.7 μm; Phenomenex, Germany) with the flow rate set to 0.5 ml/min. The flow gradient started with 85% eluent A for 0.5 min, decreasing to 50% within 1 min, and increasing back to 85% eluent A in 0.5 min. These conditions were held for 1 min. For quantification, the peak area of dexmedetomidine was further integrated and divided by the peak area of the internal standard. Calibrator samples were fitted with a least-squares fit and weighted by 1 divided by the individual peak area (1/x). The limit of quantification of dexmedetomidine was 0.01 ng/ml, and the limit of detection was 0.005 ng/ml.

#### Quantification of Cortisol

Plasma cortisol levels were measured in blood samples collected at baseline, 90, 180, 360, and 480 min after drug administration. The plasma samples were spiked with 38 µl internal standard, 250 µl ZnSO_4_, and 500 µl purified water was added. The mixture was vortexed and centrifuged at 8,000*g* for 5 min. Afterward, the samples were purified using a solid-phase extraction on an OasisPrime HLB 96-well plate using a positive pressure 96-well processor (both Waters, United Kingdom). For liquid chromatography-mass spectrometry analysis, a Vanquish ultra-high performance liquid chromatography (equipped with an ACQUITY ultra-performance liquid chromatography HSS T3 column, 100 Å, 1.8 µm, 1 mm × 100 mm column; Waters, Switzerland) was coupled to a Q Exactive Plus Orbitrap (both Thermo Fisher Scientific, Switzerland). Separation was achieved using gradient elution greater than 11 min using water and methanol both supplemented with 0.1% formic acid (all Sigma-Aldrich, Switzerland) as mobile phases. Data analysis was performed using TraceFinder 4.1 (Thermo Fisher Scientific, Switzerland). The method was validated according to international standards.^23^ Steroid hormone concentrations were calculated in nanomoles per liter.

#### Quantification of Melatonin

Plasma melatonin levels were measured in blood samples collected at baseline, 90, 180, 360, and 480 min after drug administration. Melatonin was quantified with LC-MS/MS, as previously reported elsewhere.^24^

### Polysomnography

Sleep was recorded by all-night polysomnography from 11:00 pm to 7:00 am with dedicated polysomnographic amplifiers (SIENNA ULTIMATE, EMS Biomedical, Austria). The recording montage consisted of 14 electroencephalography (EEG) electrodes according to the 10 to 20 system (Fp1, Fp2, F3, F4, Fz, C3, C4, Cz, P3, P4, Pz, O1, 02, Oz), a bipolar electrooculogram, a submental electromyogram (EMG), and a two-lead electrocardiogram. The analog signals were conditioned by a high-pass filter (EEG, 0.5 Hz; EMG, 5 Hz; electrocardiogram, 1 Hz) and a low-pass filter (EEG, 70 Hz; EMG, 100 Hz; electrocardiogram, 70 Hz), digitized and stored at the sampling frequency of 512 Hz.

#### Visual Scoring

Sleep variables were visually scored based on 30-s epochs according to the criteria of the American Academy of Sleep Medicine (Darien, Illinois).^25^ For sleep scoring, the F3-A2, C3-A2, and O1-A2 (in case of severe artifacts, F4-A1, C4-A1, and O2-A1) derivations were used. Movement- and arousal-related artifacts were visually identified and excluded from the analyses. The following sleep variables were analyzed: time in bed, time between lights-off and lights-on; total sleep time, time spent in stages N1, N2, and N3, and REM sleep; sleep efficiency, percentage of total sleep time per time in bed; sleep latency, time between lights-off and first occurrence of three consecutive epochs of N1 sleep or any other sleep stage; REM sleep latency, time between sleep onset and the first occurrence of REM sleep; and time spent in wakefulness after sleep onset, NREM sleep stages N1, N2, and N3, and REM sleep.

#### Quantitative EEG Analysis

After notch filtering, a low-pass filter with a cutoff frequency of 40 Hz and high-pass filter with a cutoff frequency of 0.5 Hz were applied. The EEG signal was then downsampled to 128 Hz, and C3 and C4 electrodes were re-referenced to the mastoids (C3-M2 and C4-M1). The filtered signal was decomposed in the frequency domain with the Welch’s averaged modified periodogram, using a 4-s Hanning window with 50% overlap, resulting in a 0.25-Hz frequency resolution. The EEG slow-wave energy for the derivations C3-M2 and C4-M1 in the first and second half of the night was computed as the cumulative sum of delta power (0.75 to 4.0 Hz) across artifact-free N2 and N3 sleep episodes. The derivation with the better signal quality was used for averaging. Slow-wave energy values were log_10_-transformed for analyses. The EEG preprocessing and computational analyses were carried out in MATLAB (R2024a, MathWorks, USA).

### State upon Awakening

#### Cortisol Awakening Response

To quantify the cortisol awakening response, we sampled saliva in each participant at 7:00 am (immediately after awakening), and at 7:15 am, 7:30 am, 7:45 am, 8:00 am, and 8:15 am. Participants were instructed to chew a cotton swab for 60 s and return it into the dedicated Salivette tube (Sarstedt, Germany). The tubes were immediately stored on ice until final storage at –80°C. Cortisol was quantified with LC-MS/MS, as reported elsewhere.^26^

#### Orthostatic Regulation

We conducted the Schellong test, also known as the Schellong maneuver, immediately after awakening (7:00 am), to assess potential carryover effects of dexmedetomidine on postawakening orthostatic regulation.^27^ This validated clinical test involves changing from a supine to a standing body position and monitoring various physiologic variables, including blood pressure and heart rate. The blood pressure and heart rate were measured at 1-min intervals, first three times in a supine and then five times in a standing position. Because of measurement errors, two assessments in the placebo condition and one assessment in the 20-µg dexmedetomidine condition needed to be excluded from the analyses. In addition, one assessment in the 20-µg dexmedetomidine condition was discontinued because the participant felt dizzy. To identify orthostatic dysregulations, the difference between the last blood pressure measurement in the supine position and the first three measurements in the upright position were calculated. A drop of 20 mmHg or greater in systolic and/or 10 mmHg or greater in diastolic blood pressure are considered orthostatic hypotension events.^28^ To document the mean blood pressure and heart rate values during the Schellong test, means across all time points per position were calculated.

#### Morning Sleep Questionnaire

We used a morning sleep questionnaire^29^ to assess subjective sleep quality, including estimated time to fall asleep, number of nocturnal awakenings, wake time after sleep onset, and visual analog scales to compare subjective sleep quality against a habitual night at home.

#### Sleep Inertia Questionnaire

The sleep inertia questionnaire^30^ was designed as a trait inventory, where participants are instructed to rate the quality of their awakening during the past week, focusing on physiologic, emotional, cognitive, and behavioral aspects. The instructions for the inventory were as follows: “On a typical morning in the past week, after you woke up, to what extent did you, for example, have trouble getting out of bed?” (with possible ratings ranging from 1, “not at all,” to 5, “all the time”).

For the current study, we adapted the original sleep inertia questionnaire to assess the acute subjective experiences of the volunteers at waking up.^26^ We rephrased the inventory instructions to gather information about the state of the waking process on the experimental morning, rather than trait information about the previous week. This allowed us to analyze possible immediate effects of the study drug. The revised instructions read as follows: “How strongly did you experience the following aspects after waking up this morning, in comparison to a typical morning last week, such as having trouble getting out of bed?” (with possible ratings from –3, “extremely less,” to 3, “extremely more”). For our purposes, the questionnaire was renamed as Acute Sleep Inertia Questionnaire and administered at 7:45 am.

#### Subjective Sleepiness

Before (at 10:40 pm) and after (at 7:15 am) each experimental night, we administered the validated Karolinska Sleepiness Scale.^31^

#### Positive and Negative Affect Schedule

At 7:30 am, we administered the Positive and Negative Affect Schedule,^32^ a questionnaire in which the participant is asked to rate the occurrence and intensity of 20 mood states (10 positive and 10 negative adjectives) on a 5-point Likert scale at the moment of the rating.

#### State-Trait Anxiety Inventory

We used the State-Trait Anxiety Inventory^33^ to assess the participants’ trait anxiety level at the screening night and the state anxiety symptoms at 7:35 am every experimental morning. Both questionnaires consist of 20 items, which are rated on 4-point Likert scales.

#### Psychomotor Vigilance Test

A total of 30 min before bedtime/drug intake and 15 min after awakening, we administered a 10-min version of the psychomotor vigilance test^34^ and analyzed the median reaction time and numbers of lapses (trials with reaction time greater than 500 ms).

### Statistical Analyses

We computed independent linear mixed-effects models, with “condition” (placebo, 20 μg dexmedetomidine, 40 μg dexmedetomidine), “time” (cortisol awakening response, 7:00 am, 7:15 am, 7:30 am, 7:45 am, 8:00 am, and 8:15 am; psychomotor vigilance test, 10:30 pm and 7:15 am) and “body position” (supine, upright) as within-participant factors, and “participant identification” as a random effect. We employed R software for all analyses (R Versions 4.3.1 and 4.3.2, R Foundation for Statistical Computing, Vienna, Austria; RStudio, Inc.; R-package “lme4” Version 1.1–34^35^). In all models, we applied normal Q-Q plots to assess normality of the residuals. Moreover, we verified the assumption of homoscedasticity and linearity using a Tukey–Anscombe plot (residuals *vs*. fitted). Variables with nonnormal distributions were log-transformed. When appropriate, we carried out *post hoc* testing using the R package “emmeans” (Version 1.8.9). We corrected the *P* values of the *post hoc* tests for multiple comparisons using Benjamini–Hochberg correction of the false discovery rate. For statistically significant and trendwise significant *post hoc* comparisons, we report the estimated difference, the 95% CI of the difference, the corrected *P* value, and the standardized effect size. We used the “eff_size” function of the “emmeans” package (version 1.8.9) to calculate the effect size values and the “conf_int” function (R package “stats”) to calculate the CI. To assess the association between dosage levels of medication and the occurrence of adverse events, we conducted Fisher exact tests (R package “stats”).

## Results

### Pharmacokinetics of Oromucosal Dexmedetomidine Intake

As illustrated in figure 1, B and C, the pharmacokinetic profiles of both orodispersible formulations were exceptionally stable. The dexmedetomidine plasma concentration data were modeled by a noncompartmental, first-order absorption, and first-order elimination pharmacokinetic model. The pharmacokinetic features of the two formulations are summarized in table 1.

**Table 1.** Pharmacokinetic Parameter Estimates during Sleep after Sublingual and Buccal Dexmedetomidine Intake

Dose	Sublingual (n = 8)		Buccal (n = 17)	
	20 µg	40 µg	20 µg	40 µg
C_max_, ng/ml	0.08 ± 0.06 (76.09)	0.12 ± 0.04 (36.42)	0.11 ± 0.02 (22.12)	0.24 ± 0.06 (26.23)
t_max_, h	1.78 ± 0.71 (40.00)	1.38 ± 0.74 (54.11)	1.29 ± 0.39 (30.52)	1.73 ± 0.42 (24.05)
t_1/2_, h	3.64 ± 0.98 (26.97)	5.92 ± 4.96 (83.78)	3.56 ± 0.78 (21.85)	3.83 ± 1.00 (26.16)
AUC_all_, ng · h/ml	0.29 ± 0.11 (38.75)	0.51 ± 0.19 (37.94)	0.49 ± 0.11 (23.47)	1.12 ± 0.20 (17.52)
AUC_inf_, ng · h/ml	0.37 ± 0.15 (39.99)	0.80 ± 0.50 (57.03)	0.67 ± 0.15 (23.62)	1.46 ± 0.36 (25.02)

Values indicate mean ± SD and coefficient of variation (in parentheses; defined as the ratio of the SD to the mean) for sublingual and buccal dexmedetomidine administration.

AUC_all_, area under the plasma concentration-time curve from time point 0 (drug intake and lights-off) to time point 9 (1 h after scheduled awakening); AUC_inf_, area under the plasma concentration-time curve from time 0 to infinity; C_max_, peak plasma concentration; t_1/2_, half-life; t_max_, time to C_max_.

The sublingual formulation (fig. 1B) reached maximum plasma concentrations of 0.08 ± 0.06 (mean ± SD) ng/ml within 1.78 ± 0.71 h for the 20-μg dose and 0.12 ± 0.04 ng/ml within 1.38 ± 0.74 h for the 40-μg dose. The estimated terminal plasma half-lives were 3.64 ± 0.98 h and 5.92 ± 4.96 h and the area under the plasma concentration-time curve were 0.29 ± 0.11 ng · h · ml^–1^ and 0.51 ± 0.19 ng · h · ml^–1^ for the 20- and 40-μg doses.

The corresponding characteristics for the 20- and 40-μg doses of the improved buccal formulation (fig. 1C) were as follows: maximal plasma concentrations 0.11 ± 0.02 ng/ml and 0.24 ± 0.06 ng/ml within 1.29 ± 0.39 h and 1.73 ± 0.42 h. The estimated terminal plasma half-lives were 3.56 ± 0.78 h and 3.83 ± 1.00 h, and the area under the plasma concentration-time curve values were 0.49 ± 0.11 ng · h · ml^–1^ and 1.12 ± 0.20 ng · h · ml^–1^. All pharmacokinetic variables exhibited an exceptionally low interindividual variation.

### Sleep Variables

#### Sublingual Orodispersible Tablet

**Fig. 2.**
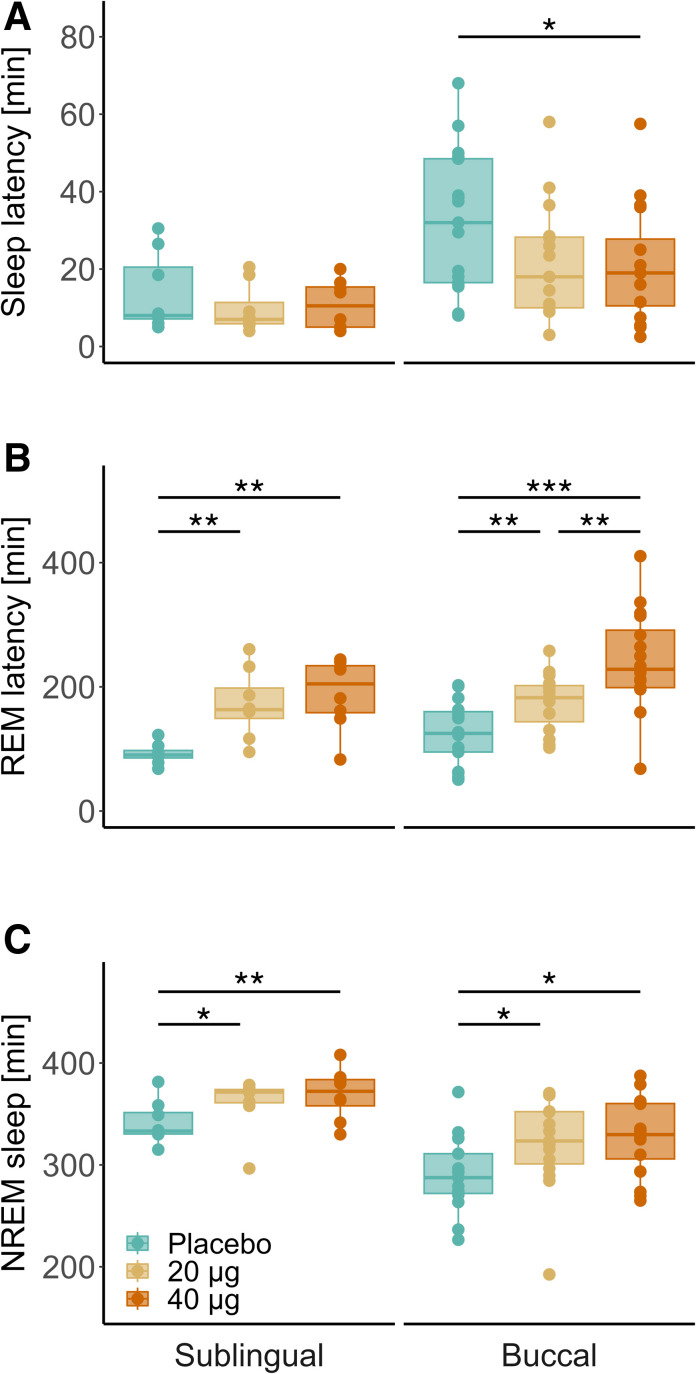
Visually scored sleep variables after intake of sublingual (*left*; n = 8) and buccal (*right*; n = 17) oromucosal dexmedetomidine formulations at bedtime. (*A*) Sleep latency until three consecutive epochs of N1 or deeper sleep stages. (*B*) Latency to first rapid eye movement (REM) sleep episode. (*C*) Total duration of non–rapid eye movement (NREM) sleep stages N2 and N3. *Box plots*: *Horizontal lines* mark the median, *lower and upper hinges* correspond to the 25th and 75th percentiles, and *whiskers* extend to the last value within 1.5 times the interquartile range. *Dots*: Individual data points. *Blue*, placebo; *yellow*, 20 µg dexmedetomidine; *orange*, 40 µg dexmedetomidine. Statistically significant differences between conditions are denoted by asterisks. *P < 0.05; **P < 0.01; ***P < 0.001 (Benjamini–Hochberg corrected).

Compared to placebo, both the 20-μg (estimate, 24.4 min [95% CI, 7.6 to 41.3 min]; *P* = 0.012; effect size = 0.87) and 40-μg (estimate, 29.0 min [12.1, 45.9 min]; *P* = 0.007; effect size = 1.04) doses of dexmedetomidine prolonged the time spent in NREM sleep (combined stages N2 and N3) and delayed the latency to REM sleep (20 μg: estimate, 80.1 min [30.1, 130.0 min], *P* = 0.006, effect size = 1.72; 40 μg: estimate, 97.8 min [47.8, 147.7 min], *P* = 0.003, effect size = 2.10; fig. 2). Both doses tended to increase the time spent in N2 sleep (20 μg: estimate, 17.8 min [1.5, 34.2 min], *P* = 0.052, effect size = 0.73; 40 μg: estimate, 18.0 [1.6, 34.4 min], *P* = 0.052, effect size = 0.74), whereas the 40-µg dose tended to reduce the duration of REM sleep (estimate, –14.7 min [–26.9, 2.5 min]; *P* = 0.065; effect size = 0.99). All other polysomnographic sleep variables remained unaffected by the drug (Supplemental Digital Content table S2, https://links.lww.com/ALN/D764).

#### Buccal Orodispersible Tablet

Compared to placebo, both the 20-µg (estimate, –9.5 min [–18.2, 0.7 min]; *P* = 0.053; effect size = –0.59) and 40-µg (estimate, –11.5 min [–20.1, –2.9 min]; *P* = 0.032; effect size = –0.71) doses of dexmedetomidine shortened sleep latency by roughly 10 to 11 min (fig. 2). Moreover, both doses (20 μg: estimate, 26.2 min [5.6, 46.8 min], *P* = 0.032, effect size = 0.78; 40 μg: estimate, 27.4 min [7.2, 47.6 min], *P* = 0.022; effect size = 0.81) prolonged the time spent in N2 sleep, as well as the combined stages N2 and N3 (20 μg: estimate, 30.0 min [5.4, 54.7 min], *P* = 0.028, effect size = 0.76; 40 μg: estimate, 37.2 min [13.1, 61.4 min], *P* = 0.011, effect size = 0.94). Dexmedetomidine dose-dependently delayed the occurrence of REM sleep (20 μg: estimate, 55.0 min [19.7, 90.4 min], *P* = 0.003, effect size = 0.90; 40 μg: estimate, 115.3 min [80.7, 149.9 min], *P* = 0.0001, effect size = 1.88), while the higher dose tended to reduce REM sleep duration (estimate, –18.6 min [–34.6, –2.7 min]; *P* = 0.071; effect size = –0.68). All other visually scored sleep variables remained unaffected by the dexmedetomidine when compared to placebo (Supplemental Digital Content table S2, https://links.lww.com/ALN/D764).

Upon confirmation that both oromucosal dexmedetomidine formulations similarly affected sleep architecture in good and poor sleepers, the subsequent analyses were restricted to the pharmacokinetically superior buccal formulation, which was studied in the poor sleepers.

### Quantitative Analysis of the EEG in NREM Sleep

**Fig. 3.**
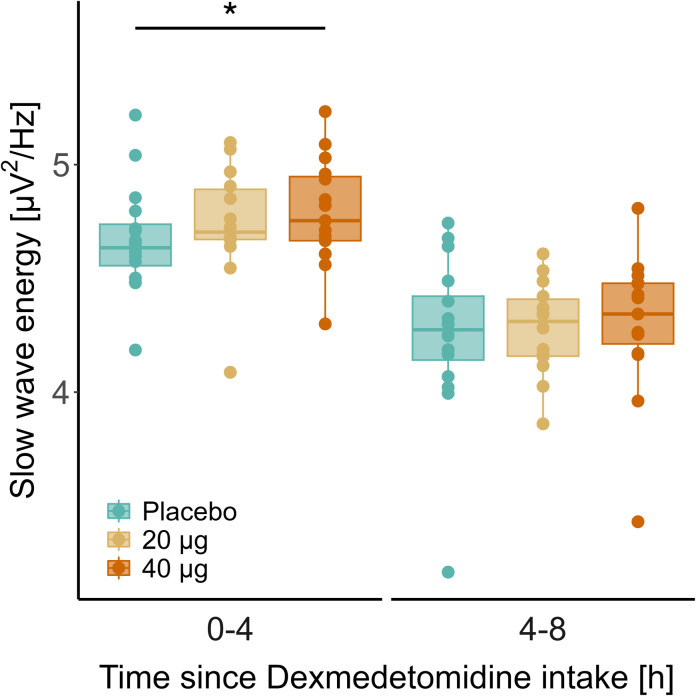
The electroencephalography slow-wave energy in the 0.75 to 4.0 Hz range in non–rapid eye movement sleep stages N2 and N3 was quantified in the first (0 to 4 h after intake) and second (4 to 8 h after intake) halves of the nights after buccal dexmedetomidine administration at bedtime. *Box plots*: *Horizontal lines* mark the median, lower, and upper hinges that correspond to the 25th and 75th percentiles, and *whiskers* extend to the last value within 1.5 times the interquartile range. *Dots*: individual data points (n = 17). *Blue*, placebo; *yellow*, 20 µg dexmedetomidine; *orange*, 40 µg dexmedetomidine. Note that the values are presented on a logarithmic scale. The asterisk indicates the significant difference between the 40 µg dexmedetomidine and placebo conditions in the first half of the experimental nights. *P < 0.03 (Benjamini–Hochberg corrected).

In all experimental nights, EEG slow-wave energy in NREM sleep decreased from the first to the second half of the sleep episodes (placebo: estimate, –0.42 [–0.56, –0.28], *P* < 0.0001, effect size = –1.55; 20 μg: estimate, –0.46 [–0.61, –0.31], *P* < 0.0001, effect size = –1.70; 40 μg: estimate, –0.49 [–0.64, –0.35], *P* < 0.0001, effect size = –1.81), reflecting the dissipation of sleep pressure (fig. 3). Compared with placebo, 40 μg dexmedetomidine increased slow-wave energy in NREM sleep in the first 4 h of the sleep episode (4.79 ± 0.24 *vs.* 4.67 ± 0.24 μV^2^/Hz; estimate, 0.09 [0.01, 0.17]; *P* = 0.03; effect size = 0.38). After intake of 20 μg dexmedetomidine, slow-wave energy tended to be higher than after placebo (4.74 ± 0.25 *vs.* 4.67 ± 0.24 μV^2^/Hz; estimate, 0.07 [–0.01, 0.16]; *P* = 0.055; effect size = 0.31). No differences among the three experimental conditions were observed in the second half of the night.

### Endocrinologic and Cardiovascular Assessments

The mean nocturnal blood plasma cortisol concentrations, the nocturnal plasma melatonin profiles, and the mean cortisol level in saliva during assessment of the cortisol awakening response did not differ between the placebo and the drug conditions (table 2). Furthermore, no differences in mean nocturnal heart rate in NREM and REM sleep were detected. The Schellong test conducted upon awakening identified very few orthostatic hypotension events that did not differ among the experimental conditions (placebo, n = 0 of 15; 20 µg, n = 2 of 15; 40 µg, n = 1 of 17). Heart rate as well as mean systolic and diastolic blood pressure in supine and upright positions revealed a normal orthostatic response and did not differ among the conditions (table 2).

**Table 2.** Endocrinologic and Cardiovascular Assessments during Sleep and upon Awakening

	Placebo	DEX_20 µg_	DEX_40__µg_	p_PLA-DEX20_	p_PLA-DEX40_	p_DEX20-DEX40_
Cortisol, nmol/l						
Mean during sleep	121.5 ± 107.3	114.8 ± 99.0	140.2 ± 125.0	0.729	0.478	0.478
Melatonin, nmol/l						
Mean during sleep	0.03 ± 0.01	0.03 ± 0.01	0.03 ± 0.01	0.876	0.876	0.876
Heart rate during sleep, beats/min						
NREM sleep	56.5 ± 6.9	55.7 ± 5.6	55.1 ± 6.8	0.978	0.301	0.301
REM sleep	58.4 ± 7.7	57.6 ± 5.7	57.9 ± 7.7	0.932	0.932	0.932
Cortisol awakening response, mmol/l						
Mean concentration	8.1 ± 4.6	8.0 ± 4.1	8.0 ± 5.1	0.873	0.873	0.873
Schellong test						
Heart rate, beats/min						
Supine	58.8 ± 9.2	60.0 ± 8.3	59.4 ± 10.5	0.948	0.948	0.948
Upright	84.2 ± 12.7*	82.1 ± 11.2*	81.8 ± 12.0*	0.730	0.730	0.904
Systolic blood pressure, mmHg						
Supine	107.7 ± 11.3	107.7 ± 12.3	105.2 ± 9.7	0.970	0.597	0.597
Upright	110.1 ± 12.6	105.6 ± 10.6	105.8 ± 13.0	0.176	0.176	0.847
Diastolic blood pressure, mmHg						
Supine	62.2 ± 7.6	60.7 ± 8.6	59.5 ± 7.4	0.503	0.406	0.604
Upright	77.2 ± 7.9*	74.4 ± 10.1*	73.0 ± 9.5*	0.392	0.274	0.578

Reported values indicate mean ± SD in healthy poor sleepers (n = 17). Cortisol and melatonin values were averaged across five nocturnal measurement points (at 0, 90, 180, 360, 480 min after lights off). Mean nocturnal heart rate was calculated across all NREM (N1, N2, and N3) and REM sleep stages between lights-off and lights-on (21:00 pm to 7:00 am). The cortisol awakening response in saliva was assessed across six measurement points (at 0, 15, 30, 45, 60, 75 min) upon scheduled awakening at 7:00 am. The mean concentration is reported. The Schellong test was initiated immediately after awakening (7:03 am to 7:11 am), and the reported values indicate means across three timepoints in the supine position and five timepoints in the upright position. The values did not differ across the experimental conditions.

* *P* < 0.001, upright *vs*. supine position.

DEX, dexmedetomidine; NREM, non–rapid eye movement; p_pla-DEX20_, comparison between placebo and 20 µg dexmedetomidine; p_pla__-__DEX40_, comparison between placebo and 40 µg dexmedetomidine; p_DEX20__-__DEX40_, comparison between 20 µg and 40 µg dexmedetomidine; REM, rapid eye movement.

### Subjective Sleep Quality, Self-rated State, and Vigilance upon Awakening

Validated questionnaires and neurobehavioral tests were employed to assess subjective sleep quality, self-rated state, and neurobehavioral vigilance upon scheduled awakening. The statistical analysis revealed no differences among the experimental conditions. The data are summarized in Supplemental Digital Content table S3 (https://links.lww.com/ALN/D765).

## Discussion

Despite accumulating preclinical and clinical evidence supporting the repurposing potential of dexmedetomidine to ameliorate stress-related sleep disturbances,^10^ no dexmedetomidine delivery system is currently available for insomnia management in outpatient settings. With this purpose in mind, we developed two oromucosal, fast-disintegrating dexmedetomidine formulations (one sublingual and one buccal) and assessed their clinical potential to enhance sleep quality at subanesthetic doses (20 and 40 µg). The pharmacokinetic analyses during sleep revealed exceptional pharmaceutical properties, particularly of buccal delivery, characterized by rapid absorption, excellent dose proportionality, and minimal variability in exposure among the study participants. The polysomnographic analyses further demonstrated the almost immediate onset of pharmacologic effects, including markedly shortened sleep latency, prolonged time spent in NREM sleep, and delayed occurrence of REM sleep. The higher dose (40 µg) of buccal dexmedetomidine also increased EEG slow-wave energy in the first half of the night. Importantly, neither of the very low doses administered affected cortisol, melatonin, and heart rate during sleep or altered cortisol secretion, cardiovascular functions, or self-rated and neurobehavioral vigilance upon awakening.

Besides IV-injectable solutions used in anesthesia (United States: Precedex^TM^; Europe: Dexdor^TM^), the U.S. Food and Drug Administration (Silver Spring, Maryland) recently approved a mucoadhesive film containing 120 to 180 µg dexmedetomidine (Igalmi^TM^, BioXcel Therapeutics, Inc., USA) to ameliorate agitation in inpatients suffering from bipolar disorder and schizophrenia. Indeed, evidence accumulates that oromucosal routes of dexmedetomidine administration exhibit reduced first-pass metabolism and lower intersubject variability in plasma concentrations than other noninvasive approaches such as oral and intranasal delivery.^17–20^ Here we demonstrate in young men that between two slightly different oromucosal dexmedetomidine formulations, buccal administration showed approximately 50% higher bioavailability, considerably lower intersubject variability in plasma exposure (coefficient of variation, approximately 20 *vs*. approximately 38), better dose proportionality, and faster onset of action than sublingual administration. We suggest that the two systems differ not only in the mucoadhesive properties of the excipients but also in saliva production and ensuing swallowing and first-pass degradation. Thus, we conclude that buccal delivery of subanesthetic dexmedetomidine doses ensures an exceptional pharmacokinetic profile that offers a precise and convenient option for outpatient administration.

To assess the effects of 20 and 40 µg dexmedetomidine on sleep, we studied two groups of healthy individuals reporting good and poor subjective sleep quality. The poor sleepers presented with subclinical insomnia (Insomnia Severity Index score between 8 and 14), but did not meet the recommended Insomnia Severity Index threshold for clinical insomnia disorder (Insomnia Severity Index greater than 14). Nevertheless, they exhibited prolonged sleep latency, longer wake time after sleep onset, reduced sleep efficiency, and shorter NREM (stages N2 and N3) sleep duration than the good sleepers (Supplemental Digital Content table S2, https://links.lww.com/ALN/D764). Both doses of dexmedetomidine hastened the onset of sleep in the poor sleepers, demonstrating a very rapid onset of action of buccal dexmedetomidine intake at bedtime. Looking at the pharmacokinetic profiles of the two studies (fig. 1), it appears that dexmedetomidine plasma levels as low as 0.05 ng/ml can promote sleep. By contrast, sleep onset remained unchanged in the good sleepers. Given their short sleep latency in the placebo condition, they probably initiated sleep in the verum nights before a relevant dexmedetomidine exposure level was present. In addition, the plasma concentration increased more slowly and reached a lower maximum with the sublingual formulation than with the buccal formulation. This difference may further explain the lack of sleep onset effects in the good sleepers.

Of note, we observed a pronounced and dose-dependent prolongation of REM sleep latency, which is consistent with previous studies.^20,36^ The prolonged and more consolidated NREM sleep characterized by increased EEG slow-wave energy in the first half of the night may have contributed to this effect. Given that a fixed sleep schedule with constant time in bed (8 h) was employed, the delayed onset of REM sleep may have contributed to the marginally reduced REM sleep duration after both sublingual and buccal 40 µg dexmedetomidine delivery (Supplemental Digital Content table S2, https://links.lww.com/ALN/D764). We suggest providing the participants in future studies with a prolonged or *ad libitum* sleep opportunity to address the question whether subanesthetic dexmedetomidine administration only delays or also reduces the expression of REM sleep.

Interestingly, EEG slow-wave energy was unchanged in the second half of the night, even though the dexmedetomidine plasma levels were still in a concentration range (0.075 to 0.15 ng/ml) that enhanced slow NREM sleep oscillations in the initial hours of sleep, particularly after intake of 40 µg dexmedetomidine. At the doses tested, dexmedetomidine may consolidate deep NREM sleep at elevated homeostatic NREM sleep pressure rather than after the dissipation of sleep propensity. In addition, the effects of dexmedetomidine on the sleep EEG may depend on the time of day of drug administration.

Further supporting this notion, subjective sleepiness was unaffected upon awakening in the morning, although a residual dexmedetomidine concentration between 0.05 and 0.1 ng/ml was still present in plasma. Conversely, dexmedetomidine plasma levels between 0.025 and 0.05 ng/ml shortened sleep latency at bedtime. Again, this finding may indicate that dexmedetomidine facilitates sleep when sleep pressure is high, rather than inducing a mere pharmacologic sedation. According to its mode of action, dexmedetomidine inhibits the activity of wake-promoting neurotransmitter systems, particularly noradrenergic and histaminergic activity originating in the locus coeruleus and the tuberomammillary nucleus.^12,37,38^ These systems are disinhibited with decreasing homeostatic sleep drive.^4,39^ Thus, when endogenous sleep propensity is low and endogenous circadian phase favors wakefulness, or in patients with elevated sympathetic activity such as during acute psychologic agitation, a higher dose of dexmedetomidine than the very low doses tested in this study may be necessary to promote sleep. In addition, we propose that buccal dexmedetomidine could be very valuable in the perioperative setting because it provides effective sedation and anxiolysis with a lower risk of respiratory depression than traditional sedatives. Its convenient administration shown here could improve patient comfort and compliance, particularly in premedication and postoperative recovery.

### Limitations

Some limitations need to be considered when evaluating the results of the current studies. First, the study samples were rather small and homogenous, consisting exclusively of healthy, young, average-weight European men. This precludes the generalization of the findings. Larger and more diverse samples are needed to better ascertain the safety, tolerability, and efficacy of this intervention in clinical populations. Second, the effects of the two dexmedetomidine formulations may not be perfectly comparable because the sublingual formulation was tested in good sleepers, while the buccal administration was tested in poor sleepers. We cannot be completely sure that the differences in the pharmacokinetic profiles are due to the different study samples, the slightly different oromucosal formulations, or a combination of these differences. Finally, to estimate the absolute bioavailability of the two formulations, IV dexmedetomidine delivery would be necessary.

### Conclusions

We developed two orodispersible galenic formulations containing 20 and 40 µg of the sympatholytic α_2_ receptor agonist dexmedetomidine and show that particularly buccal delivery has exceptional pharmacokinetic properties and exerts a very fast onset of action to promote and enhance NREM sleep in healthy poor sleepers. The intervention was safe, and we observed no orthostatic dysregulation, blunted cortisol awakening response, or impaired vigilance upon awakening. We conclude that buccal dexmedetomidine is a unique tool to elucidate the roles of the locus coeruleus–norepinephrine system in human sleep–wake regulation. Furthermore, we suggest that clinical studies are warranted to establish α_2_ receptor agonism as a novel mode of action to treat stress-related insomnia.

### Acknowledgments

The authors thank Ian Clark, Ph.D., Fabio Carbone, M.Sc., Tamara Hürlimann, M.Sc., and Andreas Maag, B.Sc., for their dedicated support in the preparation, conduct, and analysis of the study.

### Research Support

This work was supported by InnoSuisse (Bern, Switzerland; grant No. 55588.1 INNO-LS), the European Research Council (Brussels, Belgium; AdG grant No. 101055383), the Wellcome Trust (London, United Kingdom; grant No. 227100/Z/23/Z), and institutional funds.

### Competing Interests

Drs. Wespi, Dornbierer, and Landolt are listed as inventors on a pending patent on “Dexmedetomidine for the treatment of sleep disorders” (registration information: UniTectra Fall-Nr. UZ-231534a) that is assigned to University of Zurich, Zurich, Switzerland. Dr. Dornbierer declares that he cofounded Reconnect Labs, an academic spinoff company of the University of Zurich, focused on the development of dexmedetomidine-based products for the treatment of sleep disorders. Dr. Landolt has received consultation fees from Heel Biologische Heilmittel GmbH (Baden-Baden, Germany). The other authors declare no competing interests.

### Reproducible Science

Full protocol available at: landolt@pharma.uzh.ch. Raw data available at: landolt@pharma.uzh.ch.

## Supplemental Digital Content

Table S1: Demographics, https://links.lww.com/ALN/D763

Table S2: Sleep variables, https://links.lww.com/ALN/D764

Table S3: Sleep quality and state upon awakening, https://links.lww.com/ALN/D765

## Supplementary Material


