# Supplementary material for: Effects of Subanesthetic Oromucosal Dexmedetomidine on Sleep in Humans: A Randomized, Controlled Pharmacokinetics–Pharmacodynamics Study
Source: Anesthesiology. 2024 Nov 27;142(3):476–87. doi: 10.1097/ALN.0000000000005314 (PMC11801451; doi:10.1097/ALN.0000000000005314)
Supplement: Supplementary file 1 [file aln-142-476-s001.pdf]

**Supplementary Table S1.** Demographic characteristics of study participants.

| | **Healthy good sleepers** (sublingual DEX intake) | **Healthy poor sleepers** (buccal DEX intake) | **p value** |
|---|---|---|---|
| Sample size (n) | 8 | 17 | |
| Sex ratio (male / female) | 8 / 0 | 17 / 0 | |
| Age (years) | 23.1 ± 3.6 | 24.4 ± 3.4 | 0.408 |
| Height (cm) | 181.9 ± 9.5 | 176.9 ± 6.47 | 0.208 |
| Weight (kg) | 74.9 ± 9.7 | 70.0 ± 6.5 | 0.224 |
| Body-mass-index ($kg/m^2$) | 22.6 ± 2.0 | 22.4 ± 1.8 | 0.778 |
| Trait anxiety | 42.1 ± 5.1 | 45.7 ± 5.8 | 0.753 |

Values indicate means ± standard deviations. Trait anxiety: score on the State-Trait Anxiety Inventory of Spielberger [1]. The two groups did not differ in any of the reported variables as assessed using an independent samples t-test.

**Reference**

1 Spielberger, C., Gorsuch, R., Lushene, R., Vagg, P. & Jacobs, G. *Manual for the State-Trait Anxiety Inventory.* Consulting Psychologists Press (1983).
