# Supplementary material for: Effects of Subanesthetic Oromucosal Dexmedetomidine on Sleep in Humans: A Randomized, Controlled Pharmacokinetics–Pharmacodynamics Study
Source: Anesthesiology. 2024 Nov 27;142(3):476–87. doi: 10.1097/ALN.0000000000005314 (PMC11801451; doi:10.1097/ALN.0000000000005314)
Supplement: Supplementary file 2 [file aln-142-476-s002.pdf]

Supplementary Table S2. Visually scored sleep variables.

**Sublingual ODT intake | n=8 |** (Healthy good sleepers)			
**Sleep variable**	**Placebo**	**DEX$_{20\ \mu g}$**	**DEX$_{40\ \mu g}$**
Total sleep time	430.75±17.35	441.69±28.59	444.25±13.97
Sleep efficiency	90.31±3.53	91.83±5.55	92.22±2.90
Sleep latency	13.75±10.06	9.68±6.25	10.81±6.24
REM sleep latency	92.12±16.53	172.19±54.79 **	189.88±56.87 **
WASO	33.50±11.21	30.13±21.10	28.25±13.56
N1 sleep	35.69±10.36	30.75±11.59	34.88±7.07
N2 sleep	227.50±17.10	245.31±26.68 $^{(*)}$	245.50±28.05 $^{(*)}$
N3 sleep	78.19±24.9	84.81±13.65	89.19±28.58
N2 & N3 sleep	305.69±25.25	330.13±29.82 *	334.69±28.57 **
REM sleep	89.38±15.40	80.81±11.77	74.69±17.05 $^{(*)}$
**Buccal ODT intake | n=17 |** (Healthy poor sleepers)			
**Sleep variable**	**Placebo**	**DEX$_{20\ \mu g}$**	**DEX$_{40\ \mu g}$**
Total sleep time	393.12 ± 42.08	413.27 ± 44.07	406.59 ± 51.40
Sleep efficiency	81.95 ± 8.88	86.35 ± 9.32	84.73 ± 10.7
Sleep latency	32.41 ± 17.99	21.73 ± 14.98 $^{(*)}$	21.09 ± 15.03 *
REM sleep latency	125.68 ± 49.34	173.87 ± 45.75 **	242.22 ± 80.02 ***$^{, \#\#}$
WASO	54.76 ± 40.91	44.27 ± 44.06	52.47 ± 41.43
N1 sleep	30.47 ± 15.87	30.63 ± 18.58	25.59 ± 13.25
N2 sleep	204.76 ± 31.19	232.10 ± 33.67 *	231.88 ± 36.34 *
N3 sleep	85.15 ± 32.37	88.80 ± 36.27	95.72 ± 35.66
N2 & N3 sleep	289.91 ± 35.76	320.90 ± 45.66 *	327.59 ± 37.80 *
REM sleep	72.74 ± 30.16	61.73 ± 21.05	53.41 ± 28.91 $^{(*)}$

Reported values indicate means ± standard deviations in minutes, except sleep efficiency that refers to the percentage of total sleep time per time-in-bed (constant: 480 min), for the sublingual and buccal ODT formulations. Total sleep time = combined stages N1, N2, N3 and REM sleep. WASO = wakefulness after sleep onset.

*** $p < 0.001$, ** $p < 0.01$, * $p < 0.05$, (*) $p < 0.1$ (DEX vs. placebo); $^{\#\#}$ $p < 0.01$ (20 µg vs. 40 µg).
