# Supplementary material for: Effects of Subanesthetic Oromucosal Dexmedetomidine on Sleep in Humans: A Randomized, Controlled Pharmacokinetics–Pharmacodynamics Study
Source: Anesthesiology. 2024 Nov 27;142(3):476–87. doi: 10.1097/ALN.0000000000005314 (PMC11801451; doi:10.1097/ALN.0000000000005314)
Supplement: Supplementary file 3 [file aln-142-476-s003.pdf]

Supplementary Table S3. Subjective sleep quality, self-rated state and vigilance upon awakening after buccal ODT intake.

	Placebo	$DEX_{20\ \mu g}$	$DEX_{40\ \mu g}$	$p_{PLA\text{-}DEX20}$	$p_{PLA\text{-}DEX20}$	$p_{DEX20\text{-}DEX40}$
**Subjective sleep quality** (morning questionnaire)						
Sleep latency (min)	50.6±41.4	34.5±31.7	38.6±35.6	0.055	0.115	0.491
Awakenings (#)	2.9±2.3	3.1±2.4	2.9±1.7	0.932	0.932	0.932
Time awake (min)	50.8±46.5	60.2±63.1	56.8±45.6	0.852	0.852	0.852
Restless sleep (VAS)	39.7±23.7	41.5±22.0	40.4±24.6	0.931	0.931	0.931
Deep sleep (VAS)	54.9±28.0	54.1±27.8	51.3±26.5	0.947	0.947	0.947
Tired (VAS)	41.6±22.4	49.2±24.6	38.5±20.6	0.397	0.633	0.353
Good mood (VAS)	48.6±23.6	43.4±17.6	52.2±18.6	0.573	0.573	0.573
Full of energy (VAS)	49.1±22.5	44.4±20.3	42.4±23.5	0.734	0.734	0.791
Calm (VAS)	40.7±22.1	32.5±18.0	40.9±21.1	0.288	0.969	0.288
Concentrated (VAS)	58.1±17.2	56.2±18.0	58.2±24.1	0.992	0.992	0.992
**Sleep Inertia Symptoms** (Acute Sleep Inertia Questionnaire)						
Physiological	2.1±5.0	1.6±2.9	2.7±3.0	0.695	0.695	0.695
Inertia	1.8±7.2	2.0±3.7	2.8±4.6	0.925	0.925	0.925
Cognitive	3.2±4.7	1.7±2.8	1.4±4.3	0.200	0.200	0.786
Emotional	0.1±2.8	-0.3±2.73	0.1±2.0	0.865	0.942	0.865
**Subjective Sleepiness** (Karolinska Sleepiness Scale)						
Evening	5.8±2.2	5.9±1.8	5.6±1.8	0.934	0.934	0.934
Morning	5.2±2.1	5.4±2.1	5.1±2.3	0.919	0.919	0.919
**Affect** (Positive-Negative Affect Schedule)						
Positive	22.5±7.7	23.6±5.1	22.3±6.7	0.540	0.853	0.540
Negative	13.6±5.5	12.6±2.7	14.6±6.3	0.516	0.516	0.516
**State anxiety** (State-Trait Anxiety Inventory)						
Score	39.8±5.7	40.1±5.4	39.6±6.1	0.833	0.833	0.833
**Reaction time, median** (Psychomotor Vigilance Task)						
Evening (ms)	297.1±28.7	293.2±35.6	286.0±39.3	0.949	0.188	0.188
Morning (ms)	301.6±39.0	293.3±34.9	297.8±39.6	0.445	0.470	0.480
**Lapses, number** (Psychomotor Vigilance Task)						
Evening (#)	2.1±2.5	1.5±2.9	2.0±3.1	0.366	0.898	0.366
Morning (#)	2.7±3.9	2.4±3.6	3.1±4.1	0.554	0.554	0.554

Reported values indicate mean values ± standard deviations in key metrics of subjective sleep quality as well as state and vigilance upon awakening in healthy poor sleepers (n = 17). The following validated questionnaires and tests were employed: Morning questionnaire [1], Acute Sleep Inertia Questionnaire [2], Karolinska Sleepiness Scale [3], Positive and Negative Affect Scale [4], State-Trait Anxiety Inventory [5], and the Psychomotor Vigilance Task (PVT [6]). On the PVT, the median reaction (RT) and the number of lapses (trials with RT > 500 ms) are reported.

References

1 Landolt, H. P. *et al.* Serotonin-2 receptors and human sleep: Effect of a selective antagonist on EEG power spectra. *Neuropsychopharmacol* **21**, 455-466 (1999).

2 Dornbierer, D. A. *et al.* A novel bedtime pulsatile-release caffeine formula ameliorates sleep inertia symptoms immediately upon awakening. *Sci Rep-Uk* **11**, e19734, doi:10.1038/s41598-021-98376-z (2021).

3 Åkerstedt, T. & Gillberg, M. Subjective and objective sleepiness in the active individual. *International Journal of Neuroscience* **52**, 29-37 (1990).

4 Watson, D., Clark, L. A. & Tellegen, A. Development and Validation of Brief Measures of Positive and Negative Affect - the PANAS Scales. *Journal of personality and social psychology* **54**, 1063-1070, doi: 10.1037/0022-3514.54.6.1063 (1988).

5 Spielberger, C., Gorsuch, R., Lushene, R., Vagg, P. & Jacobs, G. *Manual for the State-Trait Anxiety Inventory.* Consulting Psychologists Press, 1983.

6 Dorrian, J., Rogers, N. L. & Dinges, D. F. in *Sleep Deprivation: Clinical Issues, Pharmacology and Sleep Loss Effects.* (ed. C. Kushida), pp. 39-70. Marcel Dekker, Inc. (2005).
